# Online Social Support - an Adjunct or Substitute for Traditional Social Support: Cross-Sectional Study

**DOI:** 10.2196/90751

**Published:** 2026-03-05

**Authors:** Ruthie Liang, Ariel Pollock Star, Nofar Tsur, Moshe Shmueli, Norm O'Rourke

**Affiliations:** 1 Irvington High School Irvington, NY United States; 2 Department of Epidemiology, Biostatistics, and Community Health Sciences Faculty of Health Sciences Ben-Gurion University of the Negev Be'er Sheva Israel; 3 Department of Science, Technology and Society Bar-Ilan University Ramat Gan Israel; 4 Department of Psychology Faculty of Humanities and Social Sciences Ben-Gurion University of the Negev Be'er Sheva Israel; 5 Goldman School of Medicine Faculty of Health Sciences Ben-Gurion University of the Negev Be'er Sheva Israel; 6 Multidisciplinary Center for Research on Aging Faculty of Health Sciences Ben-Gurion University of the Negev Be'er Sheva Israel

**Keywords:** life satisfaction, loneliness, online social support, social media use, social support

## Abstract

**Background:**

In contrast to all previous generations, life today is lived both in-person and online. This creates both opportunities and risks to mental health and well-being. Social interaction is no longer geographically constrained, yet the anonymity and impersonality of social media create new problems. To quote Mike Tyson (July 2020), “Social media have made y’all way too comfortable with disrespecting people and not getting punched in the face for it.”

**Objective:**

This study set out to propose and test a hypothesized model to identify both direct and indirect predictors of life satisfaction. Independent or predictor variables included social media use, loneliness, and online and traditional social support.

**Methods:**

From March 2024 to October 2024, a total of 112 adults in the United States were recruited online and proceeded to complete study questionnaires. Participants were aged 42.62 (SD 12.74) years on average, had completed an average of 15.46 (SD 3.25) years of education, and reported an average household income of US $67,005 (SD US $41,560) per year.

**Results:**

Using path analysis, we found that social media use and online social support emerged as significant, indirect predictors of life satisfaction via loneliness and traditional in-person social support (*P*<.01). In total, 39% of variance in life satisfaction was explained by this path model (*R*^2^=0.39; *P*<.01).

**Conclusions:**

Contrary to hypothesis, these findings support the *rich get richer* hypothesis regarding online social support, not the *social compensation theory*, that is, online social support appears to function as an adjunct to in-person support, not as a substitute. The results of this study need to be replicated with more diverse, larger samples, with responses collected over multiple time points.

## Introduction

### Background

As of 2025, there were 5.42 billion social media users worldwide, underscoring both the omnipresence and ever-growing impact of social media [1]. What were initially static, text-based pages have evolved to include photos, videos, and interactive live streams. Platforms such as Facebook, Instagram, and TikTok are most popular today [2]. These provide diverse opportunities for interaction, support, and social connection unconstrained by proximity or borders [3].

However, as social media have evolved and expanded, so too have concerns regarding the impact of social media on mental health [4]. Some studies have found that social media have a positive effect on mental health [5], whereas others report no or negative effects [6]. Social media use (SMU) has become such a public health concern that the US Surgeon General suggested that warning labels appear on platforms stating that social media are harmful to mental health [7]. However, Hall [8] contends that the adverse effects of social media are overstated and specific to certain populations with existing mental health conditions [9].

To date, most research examining SMU and well-being has centered on teenagers and young adults, who tend to be heavy users of social media. However, this leaves a gap as findings from research with teenagers cannot necessarily be generalized to young and older adults [10]. For this study, we identified both direct and indirect effects of social media on mental health. Our hypothesized model also examined the mediating role of online social support on life satisfaction.

### Social Media and Life Satisfaction

Associations between SMU and life satisfaction are complex and multifaceted as social media affect well-being in various ways [11]. Moreover, the effects of social media vary by platform [12] and the personal characteristics of users [9,13].

SMU has commonly been operationalized as 3 correlated facets reflecting different online behaviors and motivation: active social SMU, active nonsocial SMU, and passive SMU [14]. Active social use entails direct engagement (eg, chatting or commenting on posts). Active nonsocial use is similar but does not entail interaction with others (eg, tagging photos and posting videos). Finally, passive SMU refers to browsing and scrolling without interaction or visible footprint. These various types of SMU have both direct and indirect effects on psychological well-being [15], and their impact is both positive and negative [11].

For instance, research reports that active SMU predicts well-being, whereas passive use is associated with anxiety and psychological distress [16]. However, results regarding the effects of passive SMU on life satisfaction are inconsistent and contradictory. As reported by Godard and Holtzman [6], the impact of passive SMU may depend on how social media are used (eg, general use vs Facebook groups).

Some suggest that the effects of social media on life satisfaction are too small to warrant concern [8]. Time spent on social media does not inevitably make users sad, lonely, and depressed, nor is there clear evidence that more time spent on social media is inherently problematic [8]. However, even if weak, these effects are nonetheless statistically significant [6]. It may be that positive and negative effects of SMU cancel each other out, suggesting no impact where, instead, effects are bidirectional [10]. Multivariate analyses are needed to identify both direct and indirect effects of SMU on life satisfaction.

### Social Support and Life Satisfaction

Social support is integral to life satisfaction [17] and today may entail both online and traditional in-person support. However, online and in-person support likely affect life satisfaction in different ways. Trepte et al [18] reported that online resources provide informational support, yet traditional support is needed for emotional and instrumental assistance. As a result, only traditional social support appears to directly predict life satisfaction [18]. However, in longitudinal research, positive online interactions predict positive affect, perceived support, and life satisfaction [19].

Two theories have been proposed to describe the role and function of online social support. The *rich get richer* hypothesis contends that those with wide, in-person social networks benefit most from online social support [20]. That is, those with trusted confidants and supportive networks simply extend that support online. Online and in-person social support are assumed to be positively correlated, although only in-person support predicts psychological well-being; that is, online support is assumed to be an adjunct to in-person support.

In contrast, online social support may, instead, provide a corrective or compensatory opportunity for those with few in-person contacts and limited social networks to redress these absences. Known as *social compensation theory*, those with weak social networks benefit most from social support garnered online [20]. Those with strong, traditional social networks are less motivated to garner support online. As a result, online and in-person social support are assumed to be inversely associated, yet both are assumed to predict psychological well-being. Existing research supports the social compensation theory for those with low levels of social interaction or support [21-25]. Our hypothesized model tested this social compensation theory with an online sample of adults in the United States.

### Loneliness, SMU, and Life Satisfaction

Loneliness significantly undermines life satisfaction [26]. This entails both social isolation and emotional detachment. That is, people can still be lonely even when surrounded by others [27]. Findings regarding associations between SMU and life satisfaction are ambiguous, whereas links between SMU and loneliness are more consistent [6]. That is, SMU enables users to feel less lonely; however, this varies across platforms and users [26].

For instance, the effect of loneliness on life satisfaction is significant but indirect, mediated by SMU [28]. Similarly, the negative effects of problematic SMU on life satisfaction can be explained by loneliness [29]. Again, the type of SMU, passive vs active, determines how SMU affects loneliness [30]. In contrast, the amount of time spent on social media is largely unrelated to loneliness or life satisfaction [8]. However, differences may exist across different groups of users [31].

**Figure 1:**
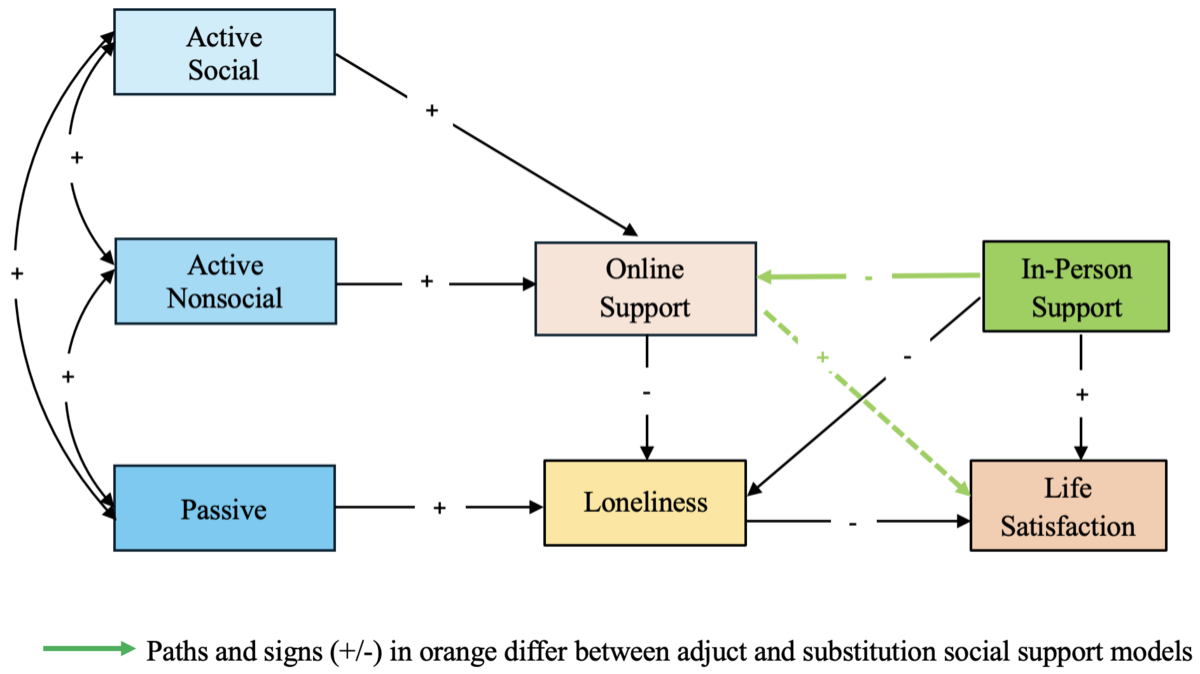
Compensatory model of online social support: a substitute for in-person support. SMU: social media use.

For this study, we applied the theory of social compensation to describe the effect of online social support on life satisfaction with an adult sample of social media users. We assumed that online and in-person support are negatively associated; that is, those with limited traditional support will foster and sustain compensatory online support networks. In addition, both online and in-person social support were assumed to directly predict life satisfaction. These effects were assumed to be both direct and indirect via lower loneliness (Figure 1).

## Methods

### Participant Recruitment

Adults aged ≥18 years were recruited as part of a larger study of SMU and well-being [32]. Prospective participants were recruited via Facebook and Instagram (Meta Inc) and the Positly platform. These platforms have previously been used to recruit participants for mental health research with general adult samples and those with psychiatric conditions [33]. For this study, we examined responses from participants with no psychiatric history but who scored within clinical range on the depression screening measure. These were participants who did not meet inclusion criteria for the central study.

Most Positly participants are Amazon Mechanical Turk contractors who have established themselves as reliable over many piecework “gigs” in the crowdsourcing marketplace [33]. Over time and multiple projects, participants provide more descriptive information (ie, psychiatric diagnoses) to gain the opportunity to take part in better-paying gigs. Positly excludes participant responses if descriptive information contradicts that previously provided or if questionnaire responses are mostly missing or determined to be unreliable (ie, rapid responding without reading the questions). Positly is designed to work with questionnaires hosted on the Qualtrics (Qualtrics International Inc) platform. Anonymized sociodemographic information is provided by Positly (eg, age and household income) along with questionnaire responses.

### Study Design

For this cross-sectional study, participants completed a series of questionnaires assessing SMU, social support, and life satisfaction. Prospective participants were first directed to an information page outlining the study and inclusion criteria. A dedicated website was created using the Qualtrics platform for secure online data collection [15,34]. Following enrollment, participants completed the instruments outlined in the following sections, along with several open-ended items regarding their use of social media.

### Ethical Considerations

Ethics approval was received from the Institutional Review Board at Ben-Gurion University of the Negev, Be’er Sheva, Israel (499-7; August 2023). Respondents provided informed consent to participate by clicking to proceed as stated on the study splash page. Participants were anonymous; they were not required to provide identifying information aside from an email address if they wished to receive a summary of findings or take part in the participant lottery (US $500). All aspects of this study were performed in accordance with the Declaration of Helsinki.

### Study Instruments

The Passive and Active Facebook Use Measure [14] was developed to study the differential effects of Facebook use. Exploratory factor analyses suggest 3 distinct patterns: active social use, active nonsocial use, and passive use. Active social use pertains to direct engagement (eg, chatting or commenting on posts), whereas active nonsocial use does not entail direct interaction with others (eg, posting videos or tagging photos), and passive use is limited to viewing photos and checking the statuses of others (ie, no engagement). Internal consistency across subscales ranges from adequate to good (0.70<Cronbach α<0.81). Construct validity for these different SMU constructs has been provided in a recent meta-analysis [6] and pilot research conducted with adults with bipolar disorder [15]. For this study, measurement was extended to general SMU by replacing the word “Facebook” with “social media” where necessary.

The brief University of California, Los Angeles Loneliness Scale (ULS-8) [35] is an abbreviated measure developed to identify associations between loneliness and health-related behaviors [36]. Responses are reported along a Likert scale ranging from “I never feel this way” (0) to “I often feel this way” (3). The internal consistency of ULS-8 responses is high (Cronbach α=0.84) [37].

The Satisfaction With Life Scale (SWLS) [38] measures perceived quality of life based on person-specific criteria [39]. The SWLS is composed of 5 questions (eg, “The conditions of my life are excellent”) with response alternatives ranging from “strongly disagree” (1) to “strongly agree” (7). Higher totals are suggestive of greater life satisfaction [40]. Test-retest reliability over 1 month has been reported as *r*=0.84 [41]. Concurrent validity of SWLS responses has been demonstrated relative to the Fordyce Global Scale (*r*=0.82) [40]. Internal consistency (Cronbach α=0.89) is strong [42].

The Brief Social Support Scale [43] measures perceived support, divided between emotional-informational support and tangible support. The reliability of the responses to both subscales is high (Cronbach α=0.87 and 0.86, respectively) [43].

The 40-item Online Social Support Scale [44] is a measure of social support received online, divided into 4 factors: instrumental (eg, financial aid, resources, and help with tasks), informational (eg, advice, help with coping, and sharing information), esteem and emotional (eg, caring, validation, and sympathy), and social companionship (eg, belonging and sharing interests and activities). Responses are reported on a Likert scale ranging from “never” (0) to “a lot” (4). Online Social Support Scale responses have shown strong internal consistency (Cronbach α=0.97) [45].

A sociodemographic questionnaire was developed for this study to obtain descriptive and psychosocial information. Participants reported their date of birth, sex, country of residence, relationship status, level of education, ethnicity, and occupation.

### Path Analysis

Path analysis was performed for this study as a 3-step process [46]. A hypothesized model was first tested (Figure 1), nonsignificant paths were deleted, and statistically significant paths not initially hypothesized were added if supported by existing research or theory.

Path analysis is an extension of linear regression, with 3 significant advantages. Path analysis allows us to simultaneously predict one or more dependent variables (indicated by the arrowhead in path models). Arrows pointing from independent to dependent variables represent significant prediction (ie, critical ratio values>|1.96|; *P*<.05). Path analysis is a multivariate statistical procedure, meaning that all significant paths emerge concurrently (ie, over and above other statistically significant results).

Path analysis allows us to identify both direct and indirect predictors of life satisfaction. Indirect prediction occurs via other variables (ie, ≥2 pathways between variables). In more complex models, variables can have direct and indirect effects on dependent variables, and indirect effects may be equal to or greater in magnitude than direct effects (total effects=direct+indirect effects).

Computing path analyses using structural equation modeling software allows us to obtain goodness-of-fit information for the overall model. Good model fit is required to interpret models [44]. In accordance with convention, we report 3 goodness-of-fit-indexes to assess overall fitness for path models: an incremental (comparative fit index; CFI), an absolute (standardized root mean square residual; SRMR), and a parsimonious (root mean square error of approximation; RMSEA) fit index. Ideal SRMR and RMSEA values are less than 0.055 [46], and ideal CFI values are greater than 0.95 [47]. Descriptive and comparative analyses were performed using SPSS (version 29; IBM Corp), and path analyses were performed using AMOS (version 29; IBM Corp).

Bootstrapping was also performed as a computationally intensive simulation approach to estimate parameters in the population [48]. Bootstrapping treats the original sample as a pseudopopulation or stand-in for population data. Multiple random samples are then drawn from the original sample along with replacement values; each is used to obtain a new set of parameter estimates. The distribution of these parameter estimates is used to compute bias-corrected CIs for path coefficients. CIs that do not cross 0 are statistically significant and likely to exist in the population [48]. Bootstrapping also allowed us to estimate statistical significance for both direct and indirect effects and compute the Bollen-Stine (BS) statistic as a further, simulation-based goodness-of-fit index.

## Results

### Descriptive Features

For this study, 112 participants were recruited between March 2024 and October 2024. The sample was almost evenly divided between men and women (n=45, 40.2% male; n=44, 39.3% female; n=13, 11.6% of the participants did not report sex). On average, participants had completed 15.46 (SD 3.25) years of education; however, household income was comparatively low given this level of education (mean US $67,005, SD US $41,560). As also reported in Table 1, the psychometric properties of responses to study instruments were largely normal and within accepted parameters.

**Table 1:** Descriptive statistics for study variables (N=112).

	Values, mean (SD)	Values, range	Skewness	Kurtosis	Cronbach α
Age (y)	42.62 (12.74)	21-84	0.75	0.17	—^a^
Education (y)	15.46 (3.25)	6-27	0.14	1.30	—
Household income (US $ per year)	67,005 (41,560)	4500-225,000	1.85	5.27	—
SMU^b^—active social use	13.60 (3.87)	5-25	0.06	0.45	0.78
SMU—active nonsocial use	7.46 (3.50)	4-20	1.43	1.95	0.87
SMU—passive use	13.71 (3.18)	4-20	–0.32	0.06	0.70
Online social support	30.58 (9.44)	12-60	0.41	0.46	0.93
In-person social support	15.31 (4.54)	6-24	0.11	–0.73	0.90
Loneliness	21.54 (5.42)	9-32	–0.34	–0.43	0.88
Life satisfaction	15.34 (7.40)	3-32	0.40	–0.82	0.90

^a^Not applicable.

^b^SMU: social media use.

### Path Analyses

We first tested the hypothesized model. Several hypothesized paths were not significant; others were added to achieve ideal fit for 2 of 3 indexes examined (*χ*^2^_11_=9.2; *P*=.30). That is, the CFI (0.99; cutoff of ≥0.95) and RMSEA (0.001; cutoff of ≤0.055) were both within ideal parameters; the SRMR was within adequate limits (0.057; cutoff of ≤0.080). With 112 participants and 6 independent variables, this model had sufficient statistical power to identify medium to large effect sizes [46].

In Table 2, we compare male and female participants. Of note, they appeared demographically similar. That is, men and women were of similar age, educational level, and average household income. Moreover, responses to the study scales were similar; only for online social support did men report significantly greater support than women.

Bootstrapping was performed to estimate bias-corrected CIs for parameter estimates, statistical significance for indirect effects, and the BS statistic. For the latter, the BS chi-square value was estimated and averaged across 500 bootstrap samples (BS *χ*^2^=10.9; *P*=.58), similar to the original sample (*χ*^2^_11_=9.2; *P*=.60). Nonsignificant chi-square values suggest generalizability of findings to the population [48].

**Figure 2:**
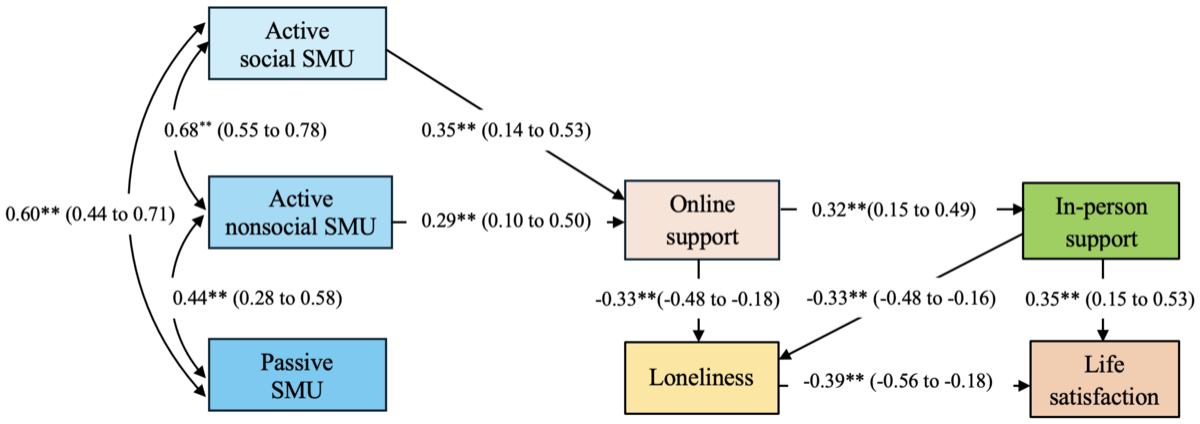
Social media use (SMU), social support, and psychological well-being. Parameter estimates are expressed as maximum likelihood estimates (standardized solution). Parenthetical numbers indicate CIs (500 bootstrapped samples). Zero is not traversed in any of the above CIs (outside of range), indicating both statistical significance and generalizability to the population. **P<.01.

As hypothesized, each of the 3 forms of SMU was positively correlated with online social support; however, their effects differed. For instance, both active social use and active nonsocial use predicted online social support; in contrast, passive SMU predicted neither loneliness nor online social support (Figure 2).

**Table 2:** Descriptive statistics for study variables: men vs women^a^.

		Male participants (n=45), mean (SD)	Female participants (n=44), mean (SD)	*t* test (*df*)
**Sociodemographic variables**				
	Age (y)	40.33 (9.52)	44.66 (13.32)	1.76 (87)
	Education (y)	15.55 (2.14)	15.47 (3.49)	0.13 (83)
	Household income (US $ per year)	65,444 (38,307)	68,602 (45,035)	0.36 (87)
**SMU^b^**				
	Active social use	13.44 (3.45)	12.93 (3.76)	0.67 (87)
	Active nonsocial use	7.76 (3.27)	6.82 (2.79)	1.45 (87)
	Passive use	13.80 (2.88)	13.20 (3.05)	0.95 (87)
**Social support**				
	Online	32.36 (7.99)	27.39 (8.04)	2.92 (87)^c^
	In-person	15.24 (4.23)	15.11 (4.42)	0.14 (87)
**Psychological well-being**				
	Loneliness	21.09 (4.99)	22.11 (5.93)	0.88 (87)
	Life satisfaction	15.64 (7.67)	13.95 (6.99)	1.09 (87)

^a^Sex not reported by 11.6% (13/112) of the participants.

^b^SMU: social media use.

^c^*P*<.01.

However, the resulting path model did not support social compensation theory as we had hypothesized. For instance, online social support and in-person social support were not inversely associated; the path coefficient between the 2 forms of social support was positive (β=0.32; *P*<.01). Nor did online social support directly predict life satisfaction; instead, the effect was indirect via both lower loneliness and greater in-person social support. Nonetheless, this indirect effect of online social support on life satisfaction was positive and statistically significant (Table 3). These findings instead support the rich get richer hypothesis. In total, 39% of variance in life satisfaction was explained by this model (*R*^2^=0.39; *P*<.01).

**Table 3:** Direct and indirect effects of social media use (SMU), social support, and well-being (N=112)^a^.

		Online social support	In-person social support		Loneliness	Life satisfaction
**Active nonsocial** **SMU**						
	Direct effects	0.29^b^		0.00	0.00	0.00
	Indirect effects	0.00		0.10^b^	–0.13^b^	0.08^b^
	Total effects	0.29^b^		0.10^b^	–0.13^b^	0.08^b^
**Active social** **SMU**						
	Direct effects	0.36^b^		0.00	0.00	0.00
	Indirect effects	0.00		0.12^b^	–0.16^b^	0.10^b^
	Total effects	0.36^b^		0.12^b^	–0.16^b^	0.10^b^
**Online social support**						
	Direct effects	—^c^		0.32^b^	–0.33^b^	0.00
	Indirect effects	—		0.00	–0.11^b^	0.28^b^
	Total effects	—		0.32^b^	–0.44^b^	0.28 ^b^
**In-person social support**						
	Direct effects	—		—	–0.33^b^	0.35^b^
	Indirect effects	—		—	0.00	0.13^b^
	Total effects	—		—	–0.33^b^	0.48^b^
**Loneliness**						
	Direct effects	—		—	—	–0.39 ^b^
	Indirect effects	—		—	—	0.00
	Total effects	—		—	—	–0.39 ^b^

^a^Statistical significance estimated over 500 bootstrapped samples.

^b^*P*<.01.

^c^Not applicable.

## Discussion

### Principal Findings

Contrary to our hypothesized model, passive SMU did not predict online social support. In addition, online social support and in-person social support appeared to be positively associated. Moreover, online social support did not directly predict life satisfaction; effects were significant but indirect. These results support the rich get richer hypothesis, as online social support appears to be an adjunct to, rather than a substitute for, traditional in-person social support. The resulting model largely emerged as hypothesized; no unhypothesized paths were added.

These findings support the assertion that online social support is distinct from traditional in-person social support [32]. Both predicted lower loneliness, but only traditional social support directly predicted life satisfaction, consistent with the rich get richer hypothesis. In addition, online social support mediated associations among SMU, loneliness, and life satisfaction. This result is consistent with previously published research reporting that associations between online social support and psychological well-being are mediated via traditional support [49].

Although a greater proportion of life is lived online today, there is no virtual substitute for traditional in-person social support [18]. Online friends cannot provide certain forms of tangible support, such as driving someone to the airport, pet sitting, or watering plants. However, this will invariably change; new programs and applications will enable online friends to provide tangible support even at a distance, a type of support that is available today only from those who are physically around us. These results are largely consistent with findings from previously published research and meta-analyses. For example, active SMU has an indirect but positive effect on life satisfaction [6]. As previously reported, associations between SMU and life satisfaction are complex; the effects of SMU on life satisfaction are similarly complex. That is, the effects of both SMU and online social support on life satisfaction appear to be mediated by loneliness and in-person social support.

Our findings do not suggest that passive SMU affects life satisfaction either directly or indirectly. Passive SMU was significantly correlated with active social and nonsocial SMU, yet passive SMU did not predict online social support, loneliness, or life satisfaction. This finding stands in contrast to those of previous research suggesting an association between passive SMU and loneliness. Moreover, we found no association between passive SMU and online social support [6]. One explanation for these conflicting findings may be the evolution of social media over time. TikTok, for instance, was only introduced in the United States in late 2018. As a result, research published before 2018 was conducted primarily or exclusively with Facebook. More photo- and video-based platforms have transformed SMU in recent years. Passive consumption of TikTok videos, for instance, may have different effects than passive use of Facebook. Accordingly, Masciantonio et al [12] contend that various social media platforms have differential effects on mental health and well-being. Instagram, for instance, appears to have an especially negative impact on teenagers and young adults [50].

### Limitations

The path model that emerged from this study allowed us to identify both direct and indirect predictors of life satisfaction. However, various limitations should be acknowledged. We did not measure personality traits, which are known to moderate social support and psychological well-being. For instance, introverted persons tend to have comparatively few acquaintances and friendships, but the latter tend to be important. Extroverts, in contrast, tend to have many acquaintances and friends. Similar personality and interpersonal differences have been found in relation to online social capital and support [51].

Differences in online support may also exist between those high and low in the trait of neuroticism [52,53]. Given their propensity for negative emotional responsivity, persons high in neuroticism seeking online social support may, instead, experience social overload, jealousy, and envy [54]. This might be due to differences in emotional regulation [55]. Research is needed to examine each of the Big Five personality traits in relation to online social support.

Future research should also be conducted with larger, more diverse samples via multiple means of recruitment. Our sample of 112 participants was sufficient for path analysis with 6 predictors; however, a larger sample size would allow us to generalize findings with greater confidence. For instance, depressive symptoms reported by study participants were high and may have confounded their responses. Our model needs to be replicated with euthymic persons (ie, those with mood within normal parameters). The findings should also be replicated using more traditional recruitment methods.

Additionally, longitudinal research is needed to identify moderating and mediating associations between SMU and well-being, and determinants of change over time. It should be stressed that the term “prediction” in path analysis is used as in multiple regression; prediction should not be conflated with causation. Replication of findings with data collected over multiple time points with diverse populations is needed to suggest causal associations. This should include cross-cultural replication and comparison to account for cultural differences in support-seeking norms and acceptance of online support.

### Conclusions

As new media technologies continue to shape social relationships, understanding their effects on mental health is essential. This study suggests that SMU influences life satisfaction largely through indirect pathways involving social support and loneliness. Online social support appears to augment rather than replace traditional in-person support. These findings support the *rich get richer* hypothesis: individuals with stronger offline networks derive greater benefit from online support. Quality of online interaction, rather than quantity of SMU, may be the critical factor in psychological well-being.

## Abbreviations

BS: Bollen-Stine
CFI: comparative fit index
RMSEA: root mean square error of approximation
SMU: social media use
SRMR: standardized root mean square residual
SWLS: Satisfaction With Life Scale
ULS-8: University of California, Los Angeles Loneliness Scale

## Data Availability

The dataset analyzed for this study is available in the Harvard Dataverse [56].
